# Multiple giant diverticula of the jejunum causing intestinal obstruction: report of a case and review of the literature

**DOI:** 10.1186/1749-7922-6-8

**Published:** 2011-03-08

**Authors:** Evangelos Falidas, Konstantinos Vlachos, Stavros Mathioulakis, Fotis Archontovasilis, Constantinos Villias

**Affiliations:** 1 First Department of General Surgery, 417 NIMTS, Veterans Hospital of Athens, 10-12 Monis Petraki, 11521, Athens, Greece; 2 First Department of Therapeutic Endoscopy and Laparoscopic Surgery, Iaso General Hospital, 264 Mesogion Avenue, 15562, Cholargos, Greece

## Abstract

Multiple diverticulosis of jejunum represents an uncommon pathology of the small bowel. The disease is usually asymptomatic and must be taken into consideration in cases of unexplained malabsorption, anemia, chronic abdominal pain or discomfort. Related complications such as diverticulitis, perforation, bleeding or intestinal obstruction appear in 10-30% of the patients increasing morbidity and mortality rates. We herein report a case of a 55 year-old man presented at the emergency department with acute abdominal pain, vomiting and fever. Preoperative radiological examination followed by laparotomy revealed multiple giant jejunal diverticula causing intestinal obstruction. We also review the literature for this uncommon disease.

## Introduction

Multiple diverticulosis of the jejunum constitutes an uncommon pathology of the small bowel. The disease is often asymptomatic and must be taken into consideration in cases of unexplained malabsorption, anemia, chronic abdominal pain and discomfort. Related complications such as diverticulitis, hemorrhage, obstruction and perforation present high mortality and morbidity rates. We herein report a case of a 55 year-old man presented at the emergency department because of acute abdominal pain, vomiting and fever. Preoperative radiological examination followed by laparotomy revealed multiple and giant jejunal diverticula causing intestinal obstruction. We also review the literature for this uncommon disease.

## Case Presentation

A 55-year old man arrived at the emergency department complaining of 48-hour lasting intense abdominal pain and vomiting. The patient had a free medical history and was not receiving any drugs at that time. He mentioned a two-year-lasting remittent abdominal pain, fullness and often abdominal distension. The patient also mentioned a particular intolerance of pulse and vegetables.

**Figure 1:**
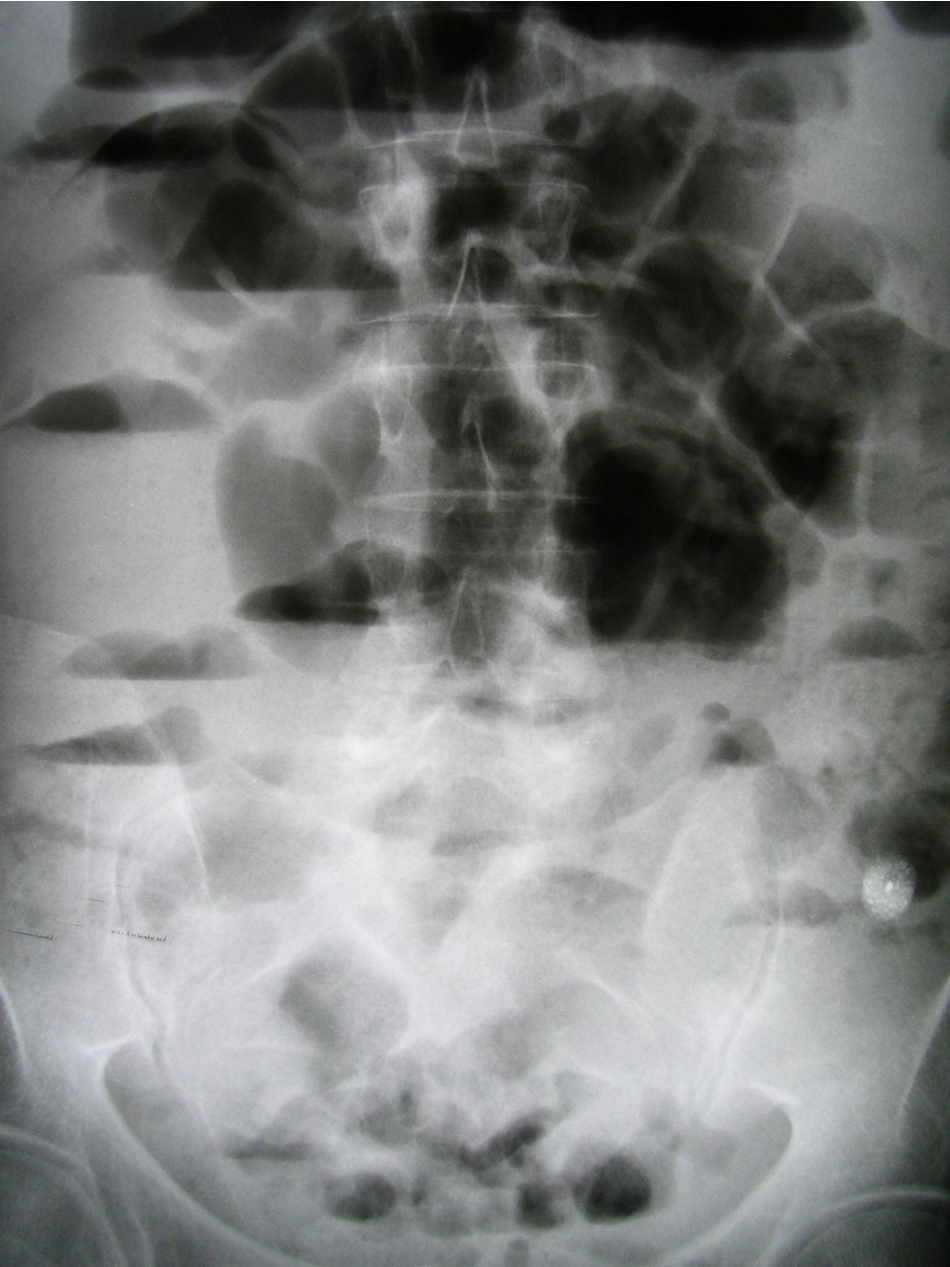
**X-ray abdominal film on admission, showing distended small bowel loops and gas-fluid levels**.

**Figure 2:**
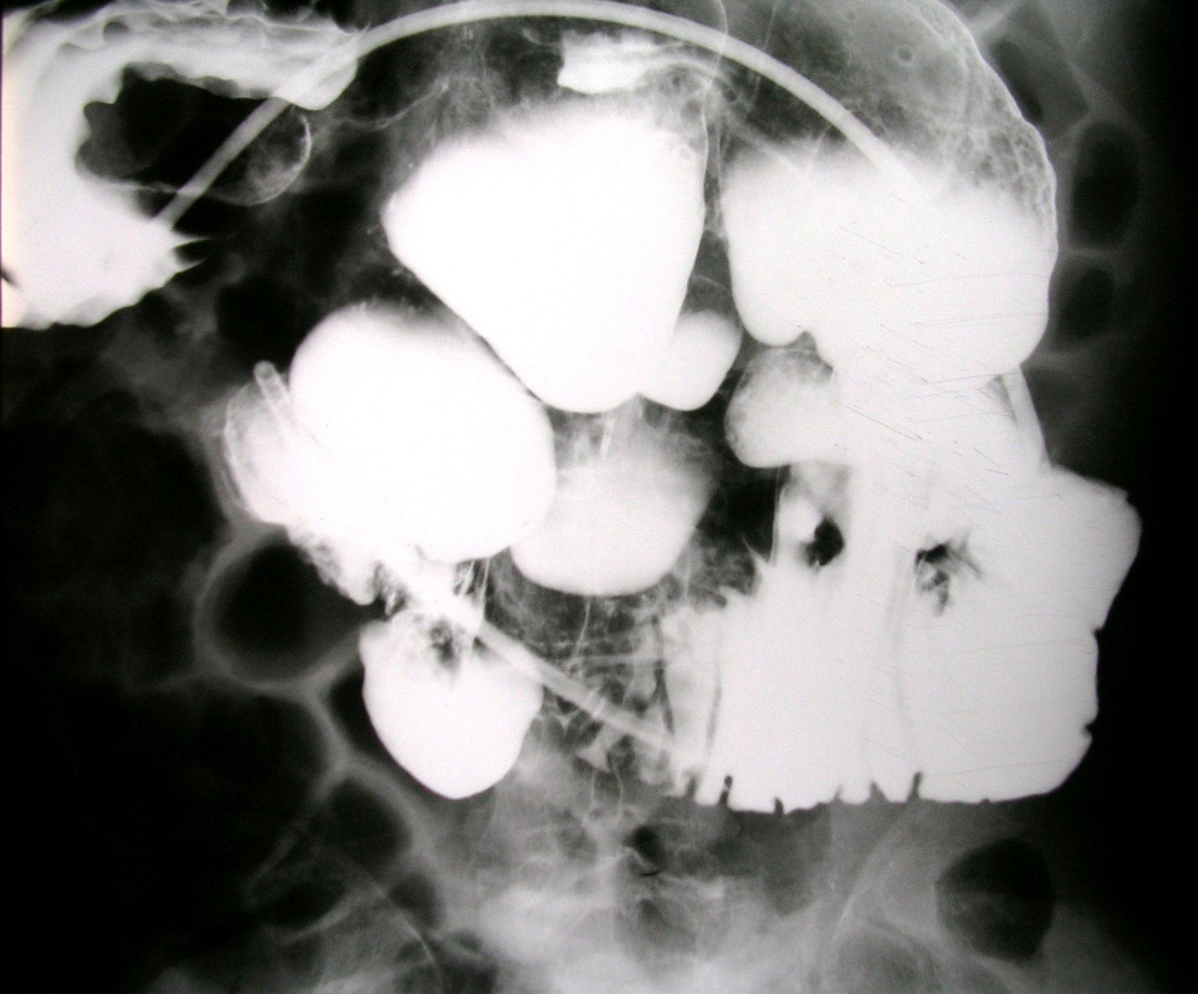
**Enteroclysis showing multiple and dilated jejunal diverticula**.

Physical examination revealed a distended abdomen with increased bowel peristalsis. Rectal examination was normal. Only his temperature was elevated (38.2°C) while other vital parameters were within normal limits. Abnormal laboratory findings included leukocytosis (13300/mm^3^), anemia (Hct:30%), hypokalemia (3.2 mmol/l) and hypoalbuminemia (2.80 mmol/l). C-reactive protein was also elevated (4.57 mg/dl). A plain abdominal X-ray showed multiple air-fluid levels and dilated intestinal loops suggesting intestinal obstruction but not signs of perforation (Figure 1). Abdominal ultrasonography revealed dilated and hyperactive intestinal loops but not free intraperitoneal fluid. Gallstones were also incidentally found. The abdominal computed tomography (CT) scan demonstrated multiple distended small bowel loops and jejunal diverticula. The patient had a nasogastric tube and received intravenously fluids, antibiotics (ciprofloxacin and metronidazole) and parenteral nutrition. Within next 72 hours, temperature and leukocytosis were decreased while the X-ray of the abdomen did not reveal gas-fluid levels. Gastroscopy and colonoscopy did not demonstrate intraluminal abnormalities while the study of the small bowel with enteroclysis showed the large and multiple jejunal diverticula (Figure 2).

**Figure 3:**
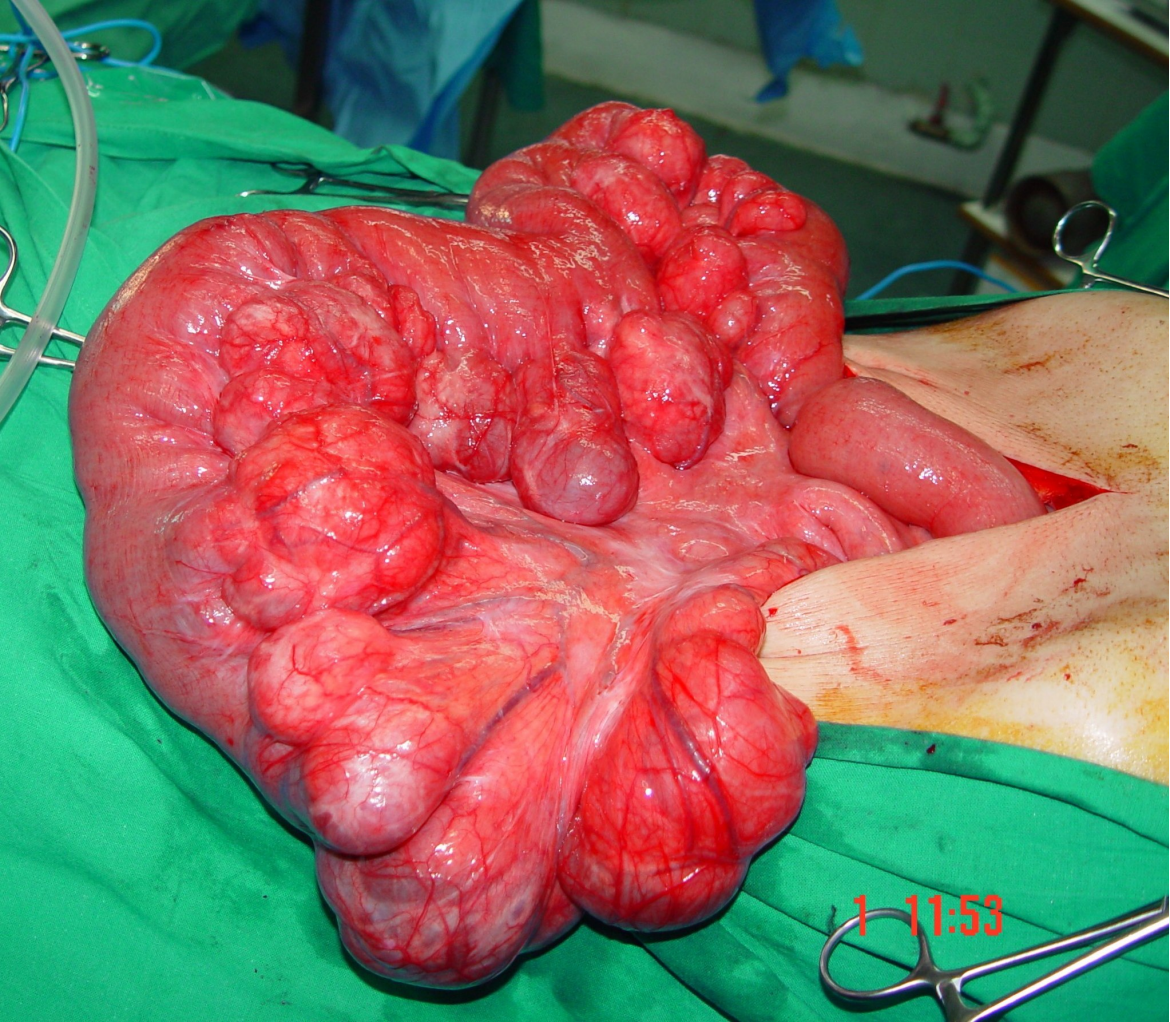
**Intraoperative findings**. Multiple giant diverticula arising at the mesenteric border of the jejunum.

**Figure 4:**
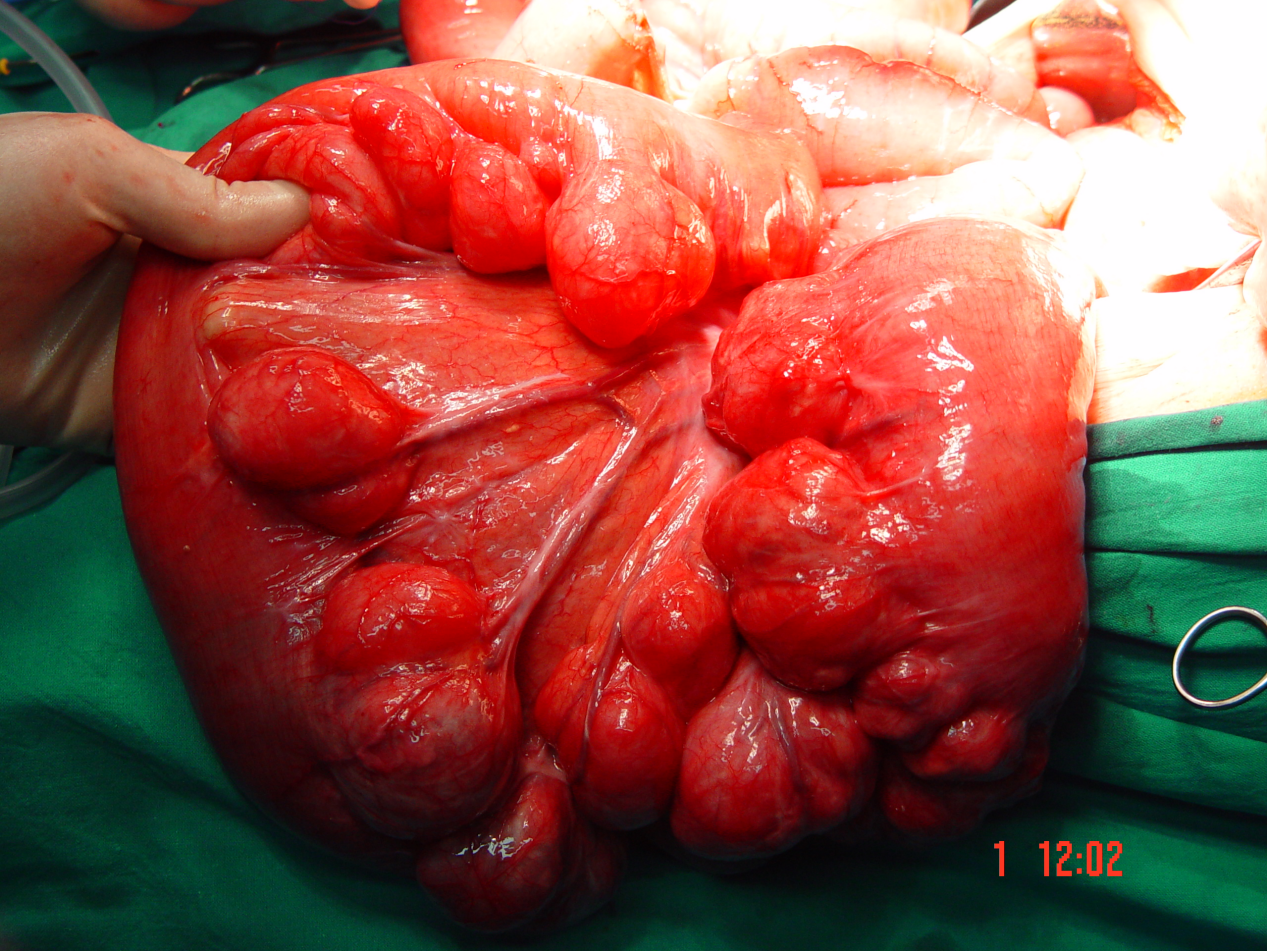
**Intraoperative findings**. Multiple giant diverticula arising at the mesenteric border of the jejunum.

The patient underwent laparotomy on day 9 after admission. Upon exploration, we found diffuse and giant jejunal diverticula with rare signs of diverticulitis (Figure 3, Figure 4). A 80 cm jejunal resection and an end-to-end anastomosis were carried out. A cholecystectomy was also performed.

The patient's post operative course was uneventful. Pathology report described large diverticula and rare focus of diverticulitis. During 24-months follow-up, the patient was symptoms free.

## Discussion

Diverticulosis of the small bowel is a rare disease with variable clinical presentations and often incidentally discovered during radiological investigations. The disease was first described by Sommering in 1794 and later by Astley Cooper in 1809. Gordinier and Shil performed the first operation for diverticula in 1906 [1,2].

Jejunoileal diverticula (excluding Meckel's diverticulum) are pseudodiverticula, resulting from a mucosal and submucosal herniation through the muscular layer of the bowels' wall in places of minor resistance to the intraluminal pressure such as the anatomic points where blood vessels penetrate the intestinal wall [2]. The etiology is unclear. Krishnamurthy et al. [3] focused on abnormalities of the smooth muscles or of the myenteric plexus in order to explain intestinal dyskinesia. Kongara et al. [4] performed manometric studies of the small bowel and described functional abnormalities in patient with small bowel diverticula. These facts support the hypothesis that irregular intestinal contractions generate increased segmental intraluminal pressure, favoring the diverticula formation through the weakest point of the bowel. A connection between intestinal diverticulosis and rare neuromuscular disorders such as Cronkhite-Canada syndrome [5], Fabry's disease [6] and mitochondrial neurogastrointestinal encephalomyopathy [7] has been described. Diffuse gastrointestinal giant diverticulosis with perforation and malabsorpion associated with giant jejunal diverticula in Elhers-Danlos syndrome have also been reported [8,9]. Progressive systemic sclerosis often involves the gastrointestinal tract and constitutes a characteristic example of proven dysmotility and acquired origin of the jejunoileal diverticulosis. Manometric studies, performed in patients with the disease, demonstrated intestinal dysmotility in 88% of the cases examined [10]. Weston et al. [11] reported an important incidence of small bowel dilation and diverticula (42%) in patients with progressive systemic sclerosis. A case of association between jejunal diverticulosis and myasthenia gravis has recently published in order to emphasize the possible correlation between anticholinesterase drugs and increased intraluminal pressure [12]. Primary or secondary amyloidosis is commonly associated with dysmotility disorders of the large and small bowel and cases of diverticular disease have been described [13-15]. Despite small bowel diverticulosis seems to be acquired, two cases of familiar predisposition have been reported [16,17].

The incidence of jejunoileal diverticula in studies of the small bowel by enteroclysis is 2-2.3% which is comparable to autopsy data presenting an incidence of 1.3-4.6% for diverticula of the jejunum and ileum [18-20]. The jejunoileal diverticulosis is usually multiple, more frequently located in the jejunum and in the terminal ileum and probably due to the larger size of the vasa recta at these areas [20]. Eighty percent of diverticula occur in the jejunum, fifteen percent in the ileum and five percent in both [1]. Isolated jejunal diverticulosis coexists with diverticula of the esophagous (2%), of the duonenum (26%) and of the colon (35%) [21]. The prevalence increases with the age and the disease presents a peak incidence at the sixth and seventh decades with a male predominance [22]. The size of small bowel diverticula varies. Diverticula may measure from few millimeters up to more than 3 cm. Performing a web search of the relative literature for giant jejunal diverticula and using terms such as 'giant jejunal divericula', 'giant jejunal diverticulosis' and 'giant jejunoileal diverticulosis', we found a limited number of cases defined from the author's description as giant; one case associated with Ehlers-Danlos Syndrome and malabsorption [8], one associated with iron deficiency [23], two cases with diverticultis [24,25], one presented with intestinal obstruction [26] and one manifested with intestinal bleeding [title only] [27]. The problem in our research was the fact that in many case reports as well as in larger series, there was no objective measurement of the size of the diverticulum (intraoperative or pathological). In many reports, the description of the diverticula was based on no medical terms (egg, golf ball etc) or it was not reported at all [28,29]. Liu et al. [30] in a series of 27 patients reported jejunoileal diverticula greater than 3 cm in 12 cases not specifying the precise size of the reported diverticula. Despite this problem, we identified a giant divericula measuring about 26 cm in a young patient with peritonitis [abstract only] [31].

The disease is usually silent. Nevertheless, Rodrigez et al. [21] reviewed the literature and noted symptoms in 29% of the cases. Many symptoms may be misdiagnosed as dyspepsia or irritable small bowel. Edwards described a symptom triad observed in these patients as 'flattulent dyspepsia' (epigastric pain, abdominal discomfort, flatulence one or two hours after meals) [32]. Vague and chronic, mainly post prandial, epigastric cramping pain, bloating or abdominal fullness is usually referred. Anemia due to iron deficiency and megaloblastic anemia have often been reported and commonly attributed to malabsorpion, steatorreia, and vitaminic deficit [23,33]. Malabsorpion could be justified by the non syncronous peristaltic movement of the bowel, the dilation of the diverticula, the stasis of the intestinal content and the bacterial overgrowth [1,34-36].

Complications such as obstruction, hemorrhage, diverticulitis and perforation occur in 10%-30% of the patients [34,35]. Some patient responds to the temporary interruption of the enteral nutrition, to a gastrointestinal relief with a nasogastric tube and to the administration of empirical, wide-spectrum antibiotics, however, complications requiring surgical intervention occur in 8-30% of patients [37,38].

Incidence of diverticulitis with or without perforation ranges from 2% to 6% [39]. Jejunoileal diverticulitis presented a high mortality rate in the past (24%), however, the mortality has been minimized because of the amelioration of the diagnostic, pharmaceutical and surgical protocols [40,41]. Perforation causes localized or diffuse peritonitis but symptoms are non specific to justify differential diagnosis, considering that other abdominal conditions present similar clinical aspects. Complications such as abdominal abscesses, fistulas and hepatic abscesses are possible [40]. Two authors described also 'microperforations' of the diverticula causing chronic, repetitive and asymptomatic pneumoperitoneum [42,43]. Diverticulitis is not always the cause of a perforation. Foreign bodies as well as abdominal trauma may also cause perforation of jejunal diverticula [44,45].

Mechanical obstruction can be caused by adhesions or stenosis due to diverticulitis, intussusception at the site of the diverticulum and volvulus of the segment containing the diverticula. In addition, sizable stones enclosed in the diverticula may apply pressure to the adjacent bowel wall or may escape from the diverticulum causing intestinal occlusion. Pseudo-obstruction, reported in 10-25% of cases, is usually associated with jejunal diverticulosis as a result of peritonitis (following diverticulitis), perforation, strangulation and incarceration of an enterolith within a diverticulum or related to the bacterial overgrowth and the visceral myopathy or neuropathy [44]. A wide, overloaded with liquid diverticulum may function as a pivot causing volvulus [40,45]. The formation of the enterolith may be de novo or around fruit seeds and vegetable material. The stone originates from biliar salts that deconiugated from the bacterial overgrowth within the diverticulum precipitate because of the more acidic pH of the jejunum [46].

Bleeding is a consequence of acute diverticulitis and due to the erosive results of the inflammation. Mucosal ulcerations compromise mesenteric vessels causing hemorrhage. Rodriguez et al., in a large series of 141 symptomatic cases, estimated bleeding in 2% [21].

Suspicion of jejunal diverticulosis is difficult and often the diagnosis is missed or delayed. Considering that jejunal diverticulusis is asymptomatic for a long time in most of the cases, diagnosis is usually made when the disease becomes symptomatic or complicated. Simple radiographs are not suggestive to make the diagnosis despite the fact that Nobles et al. [47] described a characteristic triad of clinical and radiographic findings of jejunoileal diverticulosis (abdominal pain, anemia and segmental dilatation in the epigastrium or in the left upper abdomen). In cases of complicated jejunal diverticulosis, plain abdominal X-ray series demonstrate distension of small bowel, air-fluid levels and pneumoperitoneum. Barium follow-through study and enteroclysis are more specific although their utility is limited in emergency conditions [48]. Computed tomography may show focal areas of out-pouching of the mesenteric side of the bowel, localized intestinal wall thickening due to inflammation or edema, abscesses, free abdominal fluids and pneumoperitoneum. Multi slice CT seems to be promising in diagnosing jejunoileal diverticula and appears more specific than enteroclysis concerning small bowel diseases [49]. Endoscopy does not identify diverticula but excludes other causes of obstruction or hemorrhage.

In cases of bleeding, a diagnostic and therapeutic approach with Tc^99 ^RBC and mesenteric angiography seems do be specific [48]. Upper GI endoscopy can identify diverticula to the second portion of the duodenum while double-ballon enteroscopy appear helpful in diagnosing small bowel disorders, however, emergency conditions such as obstruction or diverticulitis are significant limitations [50]. Recently, a successful double-balloon enteroscopy treatment for bleeding due to jejunal diverticulosis has been reported [51]. Wireless capsule endoscopy is a new hopeful technique for the detection of small bowel diseases, predominantly used in cases of occult intestinal bleeding. Although the presence of large diverticula is a relative contraindication to capsule endoscopy because of the possibility of the capsule's entrapment in small bowel diverticula, the application of this method in patients with isolated small bowel diverticulosis and occult intestinal bleeding should be decided with a relative prudence [52]. Laparoscopy becomes a valid diagnostic approach for complicated cases, it is rapidly convertible in laparatomy and it can function as a guide in order to avoid usefulness laparotomies. In addition, laparoscopy, précising the area of the intestinal complication, guide the surgeon to the ideal incision site on the abdominal wall, minimizing the time of the operation, the post-operative pain and the morbidity due to a larger abdominal incision [53]. A total laparoscopic treatment of sizable jejunal diverticulum has been recently reported [54].

Asymptomatic jejunoileal diverticulosis does not require intestinal resection [35]. Some authors consider that patients with chronic symptoms can be treated conservatively and when symptoms are persistent or refractory to treatment, resection is imperative [1]. Others, based on data demonstrating that jejunoileal diverticula, compared to diverticula of the duodenum, potentially will perforate and develop abscesses, recommend a more aggressive surgical approach in view of the lower post-operative risk of an elective intestinal resection [37,55]. Exploratory laparotomy and resection of affected intestinal segment with primary anastomosis is mandatory in case of perforation, abscesses and obstruction. Although, Novac et al [56] presented a case series of perforated diverticulitis treated conservatively with antibiotic administration and CT-guided drainage of abdominal abscesses. The extent of the segmental resection depends on the length of the bowel affected by diverticula. If diverticula involve a long intestinal segment, as commonly happens, the resection should be limited to the perforated or inflamed intestinal segment in order to avoid a short bowel syndrome. Other surgical approaches such as the invagination of the diverticula, the primary closure of the perforation and omental patch and the diverticulectomy should be avoided since they present high mortality rates [40,57]. One should also keep in mind that diverticula may recur in a patient undergone a segmental intestinal resection for diverticulosis since the mechanism of diverticula formation (neuropathy, myopathy etc.) still remains. Regarding enteroliths, some authors propose a manual or instrumental fragmentation of the stone and a gradual pushing of their fragments to the colon. Enterotomy or segmental resection should be reserved for complicated cases [26,46].

**Figure 5:**
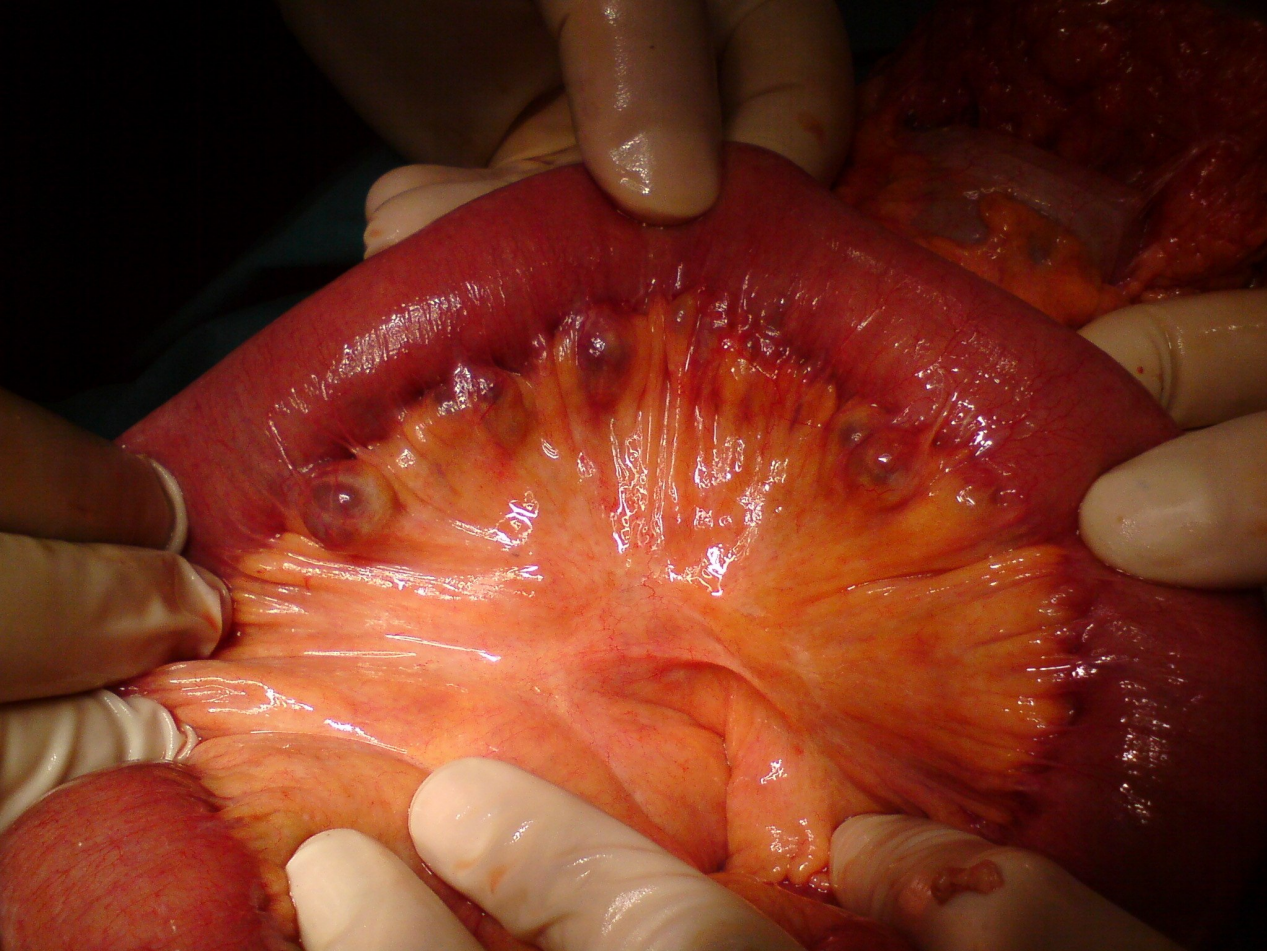
**Multiple ileal diverticula were incidentally discovered during exploratory laparotomy for intestinal bleeding**. Diverticula were not resected.

Our recent experience is limited in five cases of jejunoileal diverticulosis presented in our department in a three year period from December 2007 to December 2010. In two cases, jejunal diverticula were incidental findings during laparatomy for other reasons (colorectal cancer and multicystic hepatocarcinoma respectively). In both cases, jejunal diverticula did not present signs of inflammation or perforation and resection was not performed. In one case, clinical and imaging findings of diverticulitis suggested jejunal diverticulitis, however, the age of the patient, co-morbidities and the relative' s will led us to a conservative treatment. Bleeding was the main symptom in the fourth case and exploratory laparotomy was performed because of the ileal intraluminal entrapment of an endoscopic capsule. Bleeding was due to adenocarcinoma of the ileum and multiple small diverticula of the proximal ileum were an incidental finding (Figure 5). Divertiticula were left alone. It is important to emphasize in this case that endoscopic capsule did not described mouths of diverticula in contrast to recent reports concerning the effectiveness of the method in small bowel disorders.

In the case reported in this paper, the patient had a chronic abdominal discomfort or pain, however, he never visited the physician. The intestinal obstruction was the main symptom of presentation and obviously due to multiple overloaded jejunal diverticula and to pseudo-obstruction caused by the diverticulitis. The initial treatment with nasogastric tube and broad-spectrum antibiotics helped to limit inflammation and to avoid the extension of the diverticulitis, allowing us to perform an elective intestinal resection nine days after the initial admission. Anemia and hypoaluminemia were probably due to malabsorpion. CT scan demonstrated diverticula and excluded perforation. The enteroclysis confirmed the diagnosis.

## Conclusion

Jejunal diverticulosis is more common than reported, affects usually older patients and must be considered in the differential diagnosis in patients with acute or chronic abdominal symptoms. A high degree of suspicion is necessary in view of the high mortality and morbidity rates resulting from a delayed diagnosis of the disease. The treatment of choice is surgical excision of the affected jejunal segment.

## Consent

Written informed consent was obtained from the patient for publication of his medical data.

## Competing interests

The authors declare that they have no competing interests.

## Authors' contributions

EFand SMparticipated to the sequence alignment, researched sources for the reference and drafted the manuscript. KVLtook the photographs and drafted the manuscript. FA and CV helped in the interpretation of the photos and helped draft the final version of the manuscript.

All authors read and approved the final manuscript form.
